# Metabolic Plasticity in Schizophrenia: Clinical Rehabilitation Meets LC–MS Metabolomics and Neurofeedback

**DOI:** 10.3390/ijms27010380

**Published:** 2025-12-29

**Authors:** Mateusz Trubalski, Renata Markiewicz, Agnieszka Markiewicz-Gospodarek, Grzegorz Kalisz, Bartosz Łoza, Sylwia Szymańczyk

**Affiliations:** 1 Department of Correct, Clinical and Imaging Anatomy, Medical University of Lublin, 20-090 Lublin, Poland; mateusztrub@gmail.com (M.T.); agnieszka.markiewicz-gospodarek@umlub.edu.pl (A.M.-G.); 2 Occupational Therapy Laboratory, Medical University of Lublin, 20-093 Lublin, Poland; renata.markiewicz@umlub.edu.pl; 3 Department Bioanalytics, Medical University of Lublin, 20-090 Lublin, Poland; grzegorz.kalisz@umlub.edu.pl; 4 Department of Psychiatry, Medical University of Warsaw, 02-091 Warsaw, Poland; bartosz.loza.med@gmail.com; 5 Department of Animal Physiology, Faculty of Veterinary Medicine, University of Life Sciences in Lublin, 20-033 Lublin, Poland

**Keywords:** schizophrenia, rehabilitation, metabolomics, LC-MS, neurofeedback, neuronal plasticity, biofeedback, metabolic networks and pathways

## Abstract

Metabolomics research in schizophrenia has revealed consistent alterations across multiple biochemical domains, including energy metabolism, lipid composition, amino acid pathways, and oxidative stress regulation. The most reproducible findings include the dysregulation of the tryptophan–kynurenine pathway, disturbances in arginine/nitric oxide metabolism, alterations in phospholipid and sphingolipid profiles, reduced glutathione (GSH) in the brain, and elevated lactate levels, suggesting mitochondrial dysfunction. Antipsychotic treatment itself modifies a wide range of metabolites, complicating biomarker discovery. Although no single biomarker has yet achieved clinical utility, systematic reviews and Mendelian randomization studies provide evidence for validated biomarker panels and potential causal links between peripheral metabolite signatures and schizophrenia risk. The aim of this study is to characterize metabolic changes in patients diagnosed with schizophrenia, where each group received different non-invasive therapeutic methods and was compared to patients continuing standard pharmacotherapy without modification. The study results show that schizophrenia is associated with systemic metabolic disturbances affecting energy, amino acid, lipid, and redox pathways. Further development of research in this area requires comprehensive and long-term studies integrated with modern imaging and analytical techniques.

## 1. Introduction

Despite decades of investigation, research on schizophrenia remains largely inconclusive, even regarding the most appropriate methodological approaches [1]. The disorder’s complex and heterogeneous nature continue to challenge existing paradigms, underscoring the need for research models that integrate three critical variables: (1) the clinical state of the patient, (2) the use of valid and reliable diagnostic tools, (3) the control of treatment-related variability that may obscure biochemical interpretation.

In this exploratory study, a range of innovative research methods was applied, with particular emphasis on novel diagnostic approaches. Metabolomic analyses using liquid chromatography coupled with mass spectrometry (LC–MS) represent a modern and promising research direction aimed at identifying biochemical biomarkers for the diagnosis and therapeutic profiling of psychiatric disorders [2]. Given that many mental neuropsychiatric syndromes possess multifactorial etiologies, this line of research offers exceptional potential for advancing both pharmacotherapy and psychotherapeutic interventions [3,4].

Previous findings suggest that the search for biomarkers reflecting underlying biological processes may prove transformative—not only for disease diagnosis but also for the analysis of physiological and biochemical alterations at the cellular level. These processes are influenced by a wide range of factors, including nutrition, environmental exposure, and pharmacological treatment [4,5]. In this context, high-resolution mass spectrometry (HRMS) has become a key analytical platform. Based on accurate mass measurements (with a precision below 1 ppm), HRMS enables the structural and quantitative characterization of peptides, carbohydrates, lipids, polymers, and other metabolites in biological samples [6,7,8,9,10,11]. Among HRMS techniques, the quadrupole time-of-flight analyzer (QTOF LC/MS) is most frequently employed. It allows for the identification of ionized molecules of defined molecular mass and the determination of their concentrations in biological matrices [12]. Depending on the experimental procedure—such as metabolic profiling following drug administration, rehabilitation, or stress exposure—QTOF LC/MS can be used to identify and quantify metabolites associated with specific metabolic pathways. Notably, this method enables the detection of both extracellular and intracellular metabolites across various biofluids, including plasma, serum, urine, saliva, and cerebrospinal fluid [4].

The scientific synergy between biomarker discovery and metabolomics is of relevance to schizophrenia research, as metabolomic analyses are increasingly used to identify novel biomarkers that may inform new diagnostic criteria and therapeutic strategies. Preliminary data on metabolomic profiles and disease-specific biomarkers in schizophrenia have been reported [12,13,14,15,16]; however, the specificity of the endophenotype remains a subject of debate, and most findings have not yet been scientifically replicated. The definition of disease-specific biomarkers capable of supporting diagnosis, prognosis, and treatment monitoring, therefore, remains unresolved.

Schizophrenia, as a chronic and relapsing disorder with both genetic and neurobiological foundations, produces profound brain biochemical dysfunctions manifested by positive, negative, and general symptoms, and affects personal functioning—impairing motivation, organization, cognition, and executive control [17,18,19,20]. Classical studies of its pathophysiology have revealed multiple neurotransmission abnormalities, with earlier hypotheses focusing on dopamine, serotonin, and glutamate dysregulation [21]. Other theories emphasize alterations in aspartate, glycine, and γ-aminobutyric acid (GABA), further supporting the concept of a disrupted neurochemical balance characteristic of schizophrenia [22,23].

In contrast, metabolomic studies comparing broad biochemical panels have identified a diverse range of differential metabolites, including *tele-methylhistamine (t-MH)* [24], *N-acetyl-aspartate* [25], *α-aminobutyrate* [26], *arachidonic acid* [27], *aspartic acid* [25,26,28], *1,3-bisphosphoglycerate* [25], *cystine and eicosenoic acid* [26], *erythrose and glucuronic acid* [25], *glutathione* [27], *glycerate* [26], *glycerol* [25,28], *glycine* [25,29,30], *palmitic acid* [25,26], *3-hydroxybutyrate* [26,27], *serotonin (5-HT)* [22,27], *L-kynurenine* [27], *linoleic acid* [25,26,27], *myo-inositol* [25,26], *stearic acid* [25,26], *1-oxoproline* [31], *pentadecanoic acid* [32], *phenylalanine* [26], *serine* [26,27,29], *threonine* [27], *γ-tocopherol* [25,28], *L-tyrosine* [27], and *uric acid* [25].

He et al. [33] sought to identify a more coherent set of signaling pathways supporting the metabolomic framework of schizophrenia, reporting hybrid differences in both amino acid and lipid metabolism between treated and untreated patients compared with controls [6,9,34]. Other researchers [35] have highlighted distinct and innovative pathways related to glucose regulation and proline metabolism, demonstrating that glucose deficiency increases fatty-acid catabolism and ketone-body production, while altered amino-acid levels reflect disruptions in nitrogenous-compound biosynthesis—a process essential for cellular integrity [36,37,38]. Another biomarker of interest, identified by Koike et al. [38], is trimethylglycine (betaine), a compound involved in homocysteine metabolism through its conversion to methionine. Decreased betaine levels in schizophrenia were associated with elevated homocysteine in the brain, potentially leading to oxidative stress and neuronal damage. Additional oxidative-stress markers reported in schizophrenia include hydroxylamine, pyroglutamic acid, γ-tocopherol, and α-tocopherol [39]. Elevated hydroxylamine levels indicate increased reactive oxygen species (ROS) accumulation, while reduced tocopherol concentrations suggest weakened antioxidant defense [39,40].

Importantly, pharmacotherapy itself may fundamentally alter the biochemical and metabolomic profile of schizophrenia, thereby potentially confounding biomarker-discovery efforts [1]. In this context, clinically effective yet biochemically non-invasive neurotherapies, such as galvanic skin response (GSR) biofeedback or structured intensive rehabilitation, may represent valuable alternative or complementary strategies.

A growing body of evidence indicates that biofeedback interventions yield beneficial outcomes in patients with anxiety, depression, suicidal tendencies, bipolar disorder, and schizophrenia [24,25,41,42,43,44]. Multiple physiological and psychological theories explain the mechanisms of biofeedback therapy, including the theory of neuroplasticity, behavioral and cognitive learning models, conditioning processes, and self-regulation of neuronal activity [45,46]. Biofeedback training can thus be conceptualized as an external modulatory approach employing targeted exercises to induce changes in the structure and function of neural networks through learning and memory mechanisms—an internal adaptive process.

On the other hand, structured, intensive rehabilitation programs have also demonstrated the potential for significant clinical improvement. Such programs target various domains, including social skills training, motivation and planning capacity, cognitive training, increasingly computer-assisted training (targeting perception, attention, and reasoning), and creativity development. These programs emphasize not only the acquisition of practical skills but also the enhancement of metacognition and social problem-solving abilities, providing a balanced psychosocial rehabilitation framework in which the achievement of specific skills is not used as an inclusion or exclusion criterion [21].

## 2. Results

Members of all three groups were recruited from participants of a city day-care center. They continued their routine antipsychotic treatment (“as usual”) and typical outpatient service. The study was limited only to male participants to reduce the risk of potential gender differences in biochemical variability, which could not be corrected between relatively small groups. Moreover, PANSS results can also be influenced by gender differences [18].

All recruited patients had remained relatively stable, i.e., without active psychotic episodes for not less than 18 months. The patients cannot be treated as clinically “residual” according to ICD-10-DCR, as they were quite young, active, and multi-episodic, so they fit the pattern of episodic schizophrenia with stable or progressive development of negative symptoms in the intervals between psychotic episodes (ICD-10-DCR: F20.01/F20.02) [18]. No current suicidal risk was diagnosed.

In Table 1, the main demographic and clinical parameters were presented, with no statistical differences at the baseline (T1). Almost all the patients lived on a disability pension or other social benefits. A significant proportion of the participants smoked cigarettes: NF—13/15, REH—11/15, and CON—12/15.

During the experiments, all patients continued their former antipsychotic treatment. All subjects were administered atypical antipsychotics (amisulpride, asenapine, aripiprazole, clozapine, olanzapine, quetiapine, risperidone), and only some of them additionally received typical forms (flupenthixol, perazine, zuclopenthixol). On average, half of the study participants were subjected to monotherapy (only with atypical antipsychotics): NF group—7/15, REH group 9/15, CON group—8/15. Polytherapy was delivered with either two or more atypical antipsychotics (NF 6/15, REH 5/15, CON 5/15) or a combination of atypical and typical antipsychotics (NF 2/15, REH 1/15, CON 2/15). The Chi-squared test for monotherapy vs. polytherapy was insignificant (χ^2^ = 1.77, *p* = 0.413). None of the patients had taken anticholinergic drugs or participated in any long-term somatic treatment. A comparison of the final clinical effects of therapy at T2, i.e., over a period of 3 months, is presented in Table 2 while none of the baseline parameters at T1 differentiated the NF, REH, and CON groups, at T2 differences occurred in many domains; however, they were restricted only to the NF and REH groups. In the study the galvanic skin response (GSR) method was used in NF, which consists of two elements: the tonic electrodermal component (skin conductance level, SCL) and the phasic component (skin conductance reactions, SCR). Both parameters play a key diagnostic role in the therapy of mental disorders [21,22,23,24,25,26]. They can be used as a reference in GSR-NF to modulate the patient’s emotional state depending on the current needs. Additionally, Table 3 presents recurring metabolomic changes in schizophrenia along with key references.

Measurements were taken using the exosomatic method using direct current (DC), using electrodes placed on the index and ring fingers of the left hand, which were connected to a device presenting subsequent training modules. Tasks in individual modules were displayed on a monitor, and patients performed them according to the instructions provided. The task of the subjects performing the CENTER exercise, in which they had to bring bubbles appearing on the screen into a circle in the center of the screen, was to achieve a state of relaxation, mainly by controlling breathing and heart rate. The higher the level of relaxation the patient achieved, the faster he performed the task and could move on to the next level of the module. Training in the BALANCE module focused on developing concentration skills. Participants were tasked with maintaining the balance of a ball in the middle of a tilting board, which required maximum attention. In the INSECT module, the goal was to achieve harmony between cognitive and executive processes. The task consisted of identifying moving or hidden insects displayed on the screen and clicking on them with a mouse. The slow movement of the insects reflected the gradual achievement of internal balance during training, which made it easier for the patient to complete the task. The GSR device monitored neurophysiological changes, which, based on the electrical resistance of the skin, allowed for the assessment of the psychophysical condition of the study participants. The training time was determined by the computer program and was 5 min for the CENTER and BALANCE modules and 10 min for the INSECT module. Metabolite levels were assessed before NF training and after the 3-month program.

The NF program was enriched with structured rehabilitation activities to enable behavioral training and overexpression of neurocognitive competencies acquired during the NF sessions. NF training was supported by five main modules: social training, motivation/planning ability, cognitive training, computer-assisted training (perception, attention, reasoning), and a creativity module. The emphasis was not only on teaching skills, but also on improving metacognition and solving social problems. It was a largely balanced, psychosocial therapeutic program, and achievement of any skill was not an inclusionary or exclusionary criterion. Our rehabilitation program followed to some extent the principles of cognitive therapy developed by Wykes et al. [21], demonstrating predictive potential related to the patient’s ability to function in the community [21,29,30,31]. The program involved the entire group (N15) and was not organized hierarchically or sequentially. Its goal was to change daily routines through additional social activities, building team competencies, social role training, increasing personal acceptance, and strengthening independence. Additional activities included group classes, assertiveness training and role-playing techniques, psychotherapy, psychoeducation, cognitive training, art therapy, physiotherapy, and sports activities. At least one session of group psychotherapy or psychoeducation was held each day.

**Figure 1:**
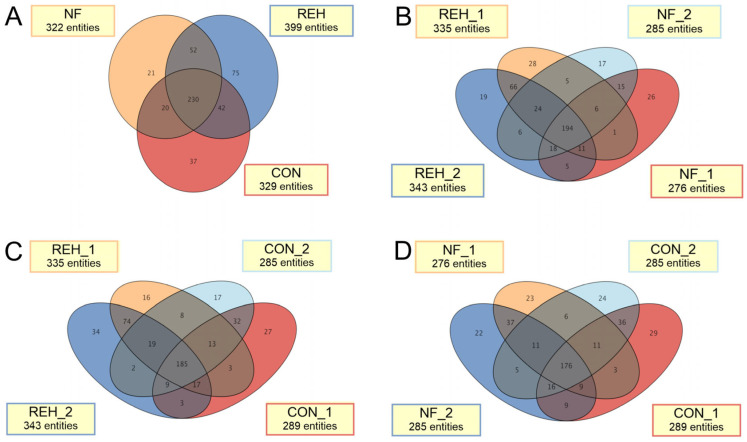
Comparative analysis of metabolites overlaps in neurofeedback (NF), rehabilitation (REH), and control (CON) groups. Three-way comparison showing shared and unique metabolites between treatment groups (**A**), four-way comparisons between initial visit and after 3 months observation results in REH vs. NF (**B**), REH vs. CON (**C**), NF vs. CON (**D**).

**Figure 2:**
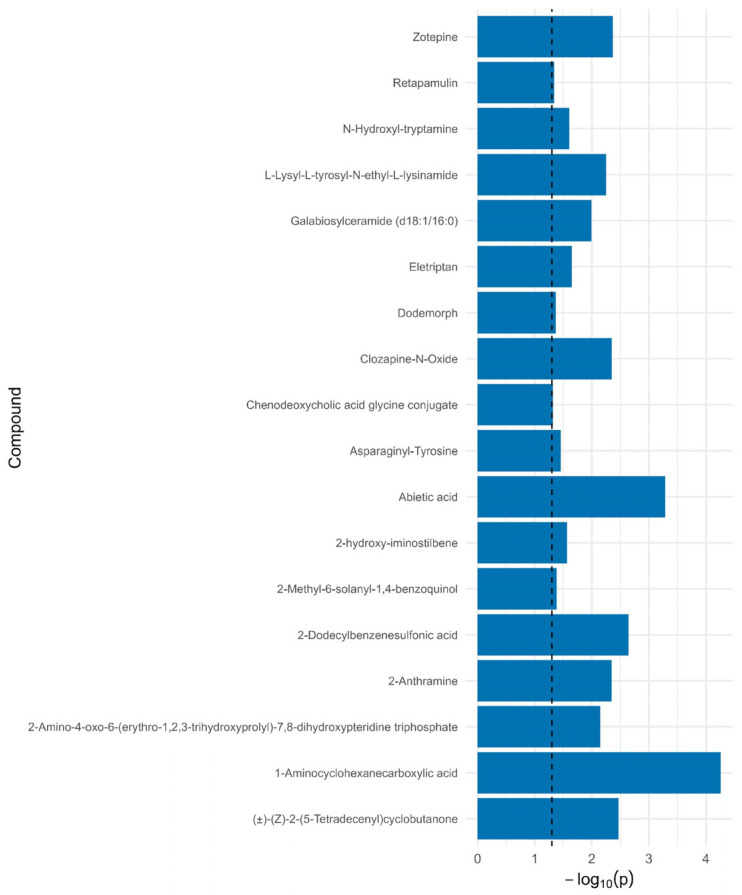
Significant metabolites differentiating, based on the time effect of groups REH, NF, and CON from two-way ANOVA. Horizontal bars represent −log_10_ (*p*-values) for each compound. The vertical dashed line indicates the threshold for statistical significance (*p* = 0.05).

**Figure 3:**
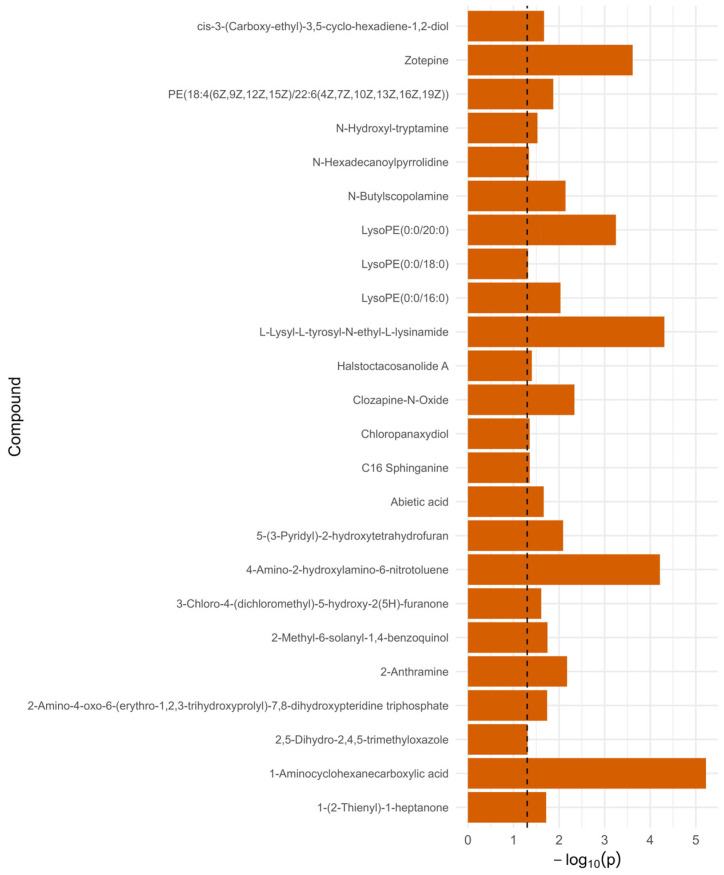
Significant metabolites differentiating, based on combined time and group interactions of groups REH, NF, and CON from two-way ANOVA. Horizontal bars represent −log_10_ (*p*-values) for each compound. The vertical dashed line indicates the threshold for statistical significance (*p* = 0.05).

Blood samples collected from patients were subjected to LC-MS analysis. Initially, in the LC-MS spectra, a total of 1042 metabolites were detected in the samples in at least one group. A two-way ANOVA was conducted for each of the 712 compounds to assess the main effects of groups (REH, NF, COM), timepoint (1 vs. 2), and their interaction. To examine metabolite detection patterns across interventions and timepoints, Venn diagrams were constructed and presented in Figure 1A, sharing 57–71% of features. The REH group showed the highest number of detected metabolites (399 entities combined across timepoints), compared with CON (329 entities) and NF (322 entities) groups. Importantly, 230 metabolites were consistently detected across all three groups (Figure 1A), representing a core metabolic signature. Group-specific metabolites (21 unique to NF, 75 unique to REH, 37 unique to CON) may represent intervention-specific metabolic responses or detection threshold effects requiring further validation. In Figure 1B–D, some temporal evolution in the metabolic profile during the intervention period was observed. Using a significance threshold of *p* < 0.05, 71 compounds exhibited significant group-related differences, 49 compounds showed significant changes over time, and 33 demonstrated significant group and timepoint interactions. Presented in Figure S1 in Supplementary Materials, the data is visualized with significant differences in Figure 1, Figure 2 and Figure 3. Accordingly, in the subsequent stages of this study, only the NF and REH groups were included in the metabolomic analysis.

Annotation against the databases indicated that many discriminating features belonged to lipid classes, primarily phosphatidylcholines, sphingomyelins, and lysophospholipids. In addition, alterations were observed in amino acid derivatives (tryptophan, arginine, and glutamate-related metabolites) and in intermediates of energy metabolism (lactate, pyruvate, and glucose-related compounds). Time-related effects were most prominent in the REH group, where several phosphatidylcholine and sphingomyelin species increased in relative abundance over the 3 months. By contrast, the NF group showed significant changes in lysophosphatidylcholines and sphingomyelins, consistent with modulation of membrane lipid turnover. The CON showed no significant changes across timepoints.

Interaction effects highlighted a subset of compounds that shifted exclusively in the intervention groups. Among these were long-chain acylcarnitines, which decreased significantly in REH, and several phosphatidylcholine species that increased selectively in NF.

**Figure 4:**
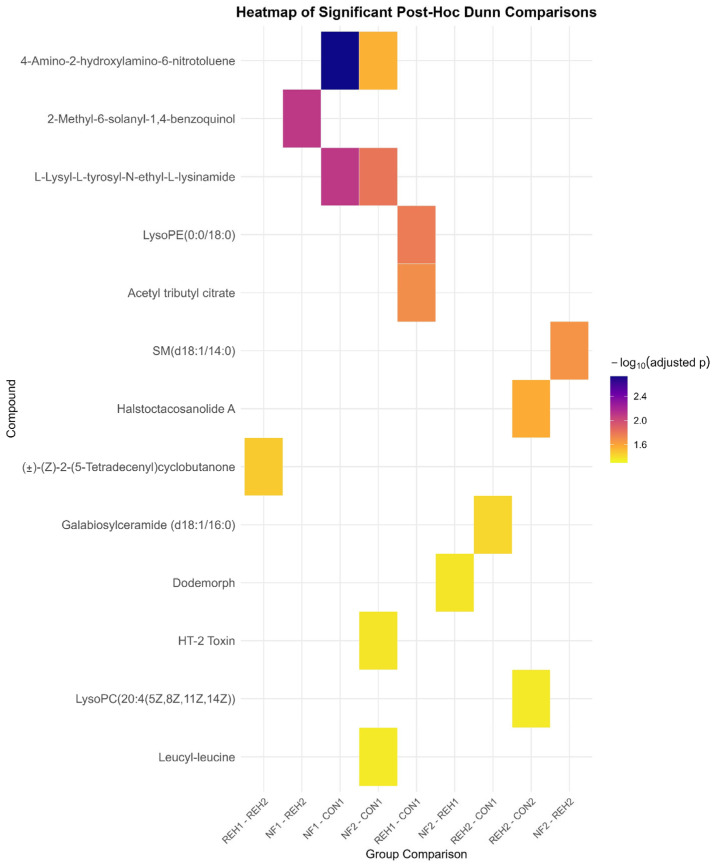
Heatmap displaying statistically significant pairwise group comparisons for individual metabolites, as identified using Dunn’s post hoc test with Holm’s correction for multiple testing (adjusted *p* < 0.05).

Pairwise comparisons were performed for each compound using Dunn’s test with Holm’s adjustment for multiple comparisons and presented in Figure 4. A heatmap was generated to display adjusted *p*-values (*p*.adj) for pairwise comparisons for identified compounds with significant differences and presented in Table 2.

The heatmap color-coded *p*-values, highlighting the significant results (*p* < 0.05) to identify meaningful differences across conditions.

## 3. Discussion

Metabolomics was used in various aspects of therapy evaluation, focusing on several aspects of human metabolism.

**Figure 5:**
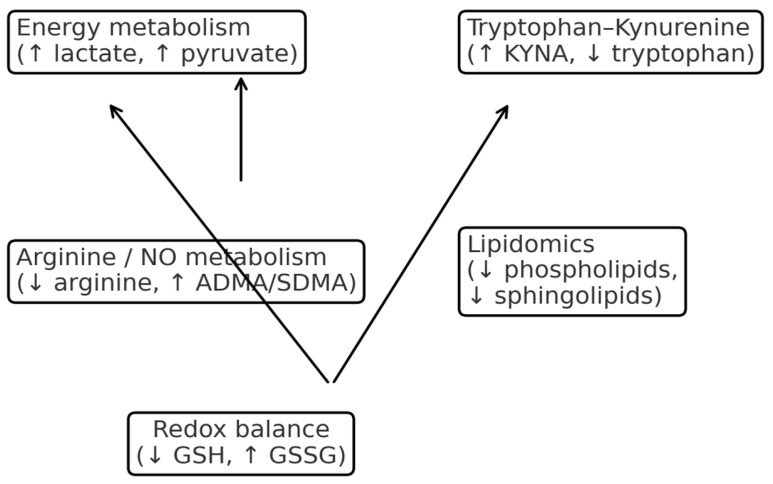
Overview of metabolite alterations by pathway (highlighting ↑ lactate/pyruvate, ↑ KYNA, ↓ arginine and ↑ ADMA/SDMA, ↓ GSH and ↑ GSSG, ↓ lipids [3,4,5,6,7,10,11,14]). ↑—increase, ↓—decrease.

Energy metabolism alterations in schizophrenia include elevated plasma and brain lactate levels, which correlate with poorer cognitive performance and are consistent with mitochondrial dysfunction [3]. Disrupted pyruvate metabolism and reduced oxidative phosphorylation efficiency have also been described [10]. Postmortem lipidomic analyses revealed reduced mitochondrial lipid signatures in white matter [11]. Within the tryptophan–kynurenine pathway, elevated kynurenic acid (KYNA) levels have been reported in first-episode psychosis, correlating with symptom severity and treatment response [4], while region-specific changes in kynurenine metabolites have been observed in brain tissue [12]. Arginine and nitric oxide metabolism are also disturbed, with studies reporting reduced arginine, elevated asymmetric dimethylarginine (ADMA)/symmetric dimethylarginine (SDMA), and altered citrulline/ornithine ratios, implicating nitric oxide synthase dysfunction and nitrosative stress [5]. Lipidomic studies have consistently shown reductions in phosphatidylcholine, phosphatidylethanolamine, and sphingolipids [6], with myelin- and mitochondria-related lipid abnormalities identified in white matter [11]. These lipid changes correlate with cognitive impairment and negative symptoms [13]. Redox and oxidative stress imbalance is evident through reduced glutathione (GSH) levels confirmed by magnetic resonance spectroscopy (MRS) meta-analysis, while elevated oxidized glutathione (GSSG) and reduced GSH/GSSG ratios predict treatment response [14]. Systematic reviews have identified reproducible metabolomic biomarkers, including decreased arachidonic acid, arginine, and aspartate, along with increased glucose-6-phosphate [2]. Mendelian randomization analyses revealed potential causal links between specific metabolites and schizophrenia risk (8), while blood metabolites such as cortisol, glutamate, and lactate have been associated with brain morphology and symptom domains [13]. Antipsychotic medications significantly affect metabolic profiles, influencing at least 14 metabolites and 12 lipid classes [8], highlighting medication as a major confounder in metabolomic studies. Despite this, metabolomics offers practical clinical applications. Diagnostic panels incorporating decreased arginine, aspartate, and arachidonic acid alongside increased glucose-6-phosphate may differentiate patients with schizophrenia from controls [2], while Mendelian randomization supports causal roles for specific metabolite ratios [9]. Prognostically, altered glutathione redox status predicts clinical outcome and electroconvulsive therapy responsiveness [14], and elevated KYNA levels in first-episode psychosis have been linked to treatment response [4]. Metabolomics also enables monitoring of metabolic shifts induced by antipsychotic treatment, offering potential for assessing therapeutic efficacy and side effect risk [8]. Finally, therapeutic strategies targeting metabolic dysregulation include glutathione restoration [7], modulation of the kynurenine pathway [12], and lipid-based interventions [6]. The main metabolomic alterations in schizophrenia are summarized in Table 3, with an overview of pathway-specific metabolite changes illustrated in Figure 5.

**Figure 6:**
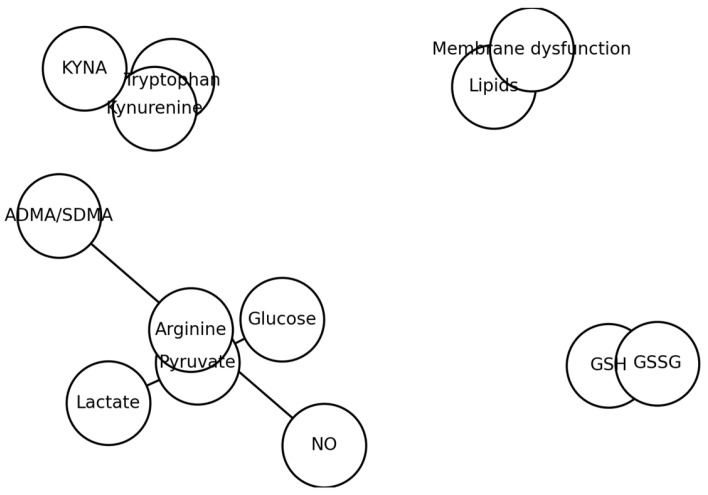
Network map of metabolic alterations in schizophrenia.

A biochemical “network map” of metabolic alterations in schizophrenia is presented in Figure 6.

The clustering of metabolites presented in this manner agrees with previous reports [3,8], ↑ KYNA [4], ↓ arginine and ↑ ADMA/SDMA [5], ↓ GSH and ↑ GSSG [7,14], ↓ lipids [5,10]. Referring to the figures presented earlier, the emerging metabolic network—derived from our study and supported by other reports—appears to be multi-layered. At its foundation lie some basic processes such as redox GSG/GSSG turnover, followed by damage-related factors (e.g., nitric oxide), and extending further to broader aspects of cellular metabolism, such as lipid pathways. This framework can thus be visualized with successive levels ranging from the fundamental mechanisms of cellular injury and, ultimately, to global metabolic imbalance. However, the specificity of metabolomic alterations is questioned, as many changes overlap with other psychiatric and neurological disorders [2].

Importantly, our data suggest that structured rehabilitation and neurofeedback interventions can modulate pathways of lipid metabolism, particularly phosphatidylcholines, sphingomyelins, and lysophospholipids, highlighting their potential responsiveness to non-pharmacological therapy. Lipid alterations have been reported in schizophrenia, and our results extend this evidence by demonstrating that structured rehabilitation and neurofeedback interventions are associated with measurable changes in peripheral lipid profiles. Our findings suggest that lipid pathways are not only markers of disease state but also sensitive to therapeutic modulation.

Antipsychotic medications significantly confound metabolic profiles [8]. For example, the false positive signal of zotepine in the presented analyses is possibly attributable to structural similarity with olanzapine and quetiapine metabolites. The actual presence of zotepine can be categorically excluded, even because of accidental inclusion. Peripheral (blood) markers sometimes fail to match central (brain) metabolomics [11,13]. Mendelian randomization suggests causal links, but most associations remain correlational [9]. Despite advances, no biomarker has achieved sufficient reproducibility for clinical diagnostics [31,47]. Heterogeneity across cohorts and platforms, as well as the schizophrenia concept itself, contributes to inconsistent findings [6,48]. The detection of acylcarnitine alterations in the REH group further supports the involvement of energy metabolism and mitochondrial processes. Reduced acylcarnitines have been interpreted as indicative of more efficient fatty acid utilization, which could represent a shift toward improved balance [48,49]. Similarly, the observation of changes in glycolytic intermediates such as lactate and pyruvate is consistent with disturbed energy metabolism in included patients [50]. However, the observed alterations in amino acids were detectable in serum metabolomics, particular pathways reported in the literature (e.g., kynurenine pathway).

As most researchers agree that only further studies in schizophrenia will allow its true significance to be evaluated, the present study was undertaken to address this need. The presented results indicate that metabolomics can capture therapy-induced biochemical plasticity in schizophrenia with biological meaning.

## 4. Materials and Methods

### 4.1. Study Design

This was a 3-month randomized, controlled trial reported using the Consolidated Standards of Reporting Trials (CONSORT) guidelines [15]. It was also registered in the ISRCTN (International Standard Randomized Controlled Trial Number) registry (study ID (Identifier): ISRCTN78612833), where the full protocol is available. Forty-five male patients with paranoid schizophrenia (according to ICD-10-DCR-International Statistical Classification of Diseased and Related Health Problems, 10th Revision, Diagnostic Criteria for Research) [16] were divided into three groups: a group in an intensive rehabilitation program (REH, rehabilitation, N = 15), a control group with standard social support and no rehabilitative interventions (CON, control, N = 15), and a group with GSR biofeedback therapy (NF, neurofeedback, N = 15). Members of all groups were recruited from among participants in the city’s day care center program. They continued antipsychotic treatment and usual clinical management. The inclusion criteria were patients’ consent, male gender, clinical diagnosis of paranoid schizophrenia [ICD], age 18–50, right-handedness (writing), no current neurological diseases, intellectual disability, nor alcohol and/or psychoactive substance addiction. The exclusion criterion was a not stable state (psychotic episodes, suicidal risk).

### 4.2. Ethical Issues

This study was conducted in accordance with the Declaration of Helsinki and approved by the Bioethics Committee at the Medical University, Al. Racławickie St. 1, Lublin, Poland, consent no KE 0254/35/2016, dated 25 February 2016. All participants in the study gave their informed consent to participate in the experiment, in accordance with the study protocol and requirements.

### 4.3. PANSS

Clinical parameters were examined with the Positive and Negative Syndrome Scale (PANSS) [21]. This 30-item interview was conceived as an operationalized instrument that provides a balanced representation of positive, negative, and general psychopathology in patients with schizophrenia. It consists of three subscales and a total score of psychopathological severity.

### 4.4. Rehabilitation Therapy

The primary aim of the intervention was to improve the social competence of the patients. The program was administered to groups and was not hierarchically or sequentially organized. It was aimed at changing the daily routine by means of additional social activities, building team competencies, training social roles, increasing personal acceptance, and strengthening one’s independence. Structured activities were held for up to 8 h daily (except at weekends). The general plan of the day included group activities such as assertive training and role-playing techniques, psychotherapy, psychoeducation, cognitive training, art therapy, physiotherapy, sports, social events, cooking meals together, entertainment activities, and relaxation training. At least one session of group psychotherapy or psychoeducation was held every day.

### 4.5. Neurofeedback Therapy (GSR Biofeedback, Galvanic Skin Response Biofeedback)

The neurofeedback (NF) training sessions were held twice a week for three months. The training plan was based on a gradual increase in the level of difficulty of the tasks, adapted to the individual progress of the participants. The galvanic skin response (GSR) method was used, which consists of two elements: the tonic electrodermal component (skin conductance level, SCL) and the phasic component (skin conductance reactions, SCR). The GSR-NF training sessions were conducted in the CENTER (relaxation), BALANCE (concentration), and INSECTS (self-control) modules using a Digi-Track apparatus (EEG-Digi Track Biofeedback- EEG/SpO2/HR, Elmiko, Poland). NF training sessions were performed in accordance with the approved schedule; training was conducted in a sound-proof room always at a specified time, mainly after breakfast. Patients were asked to avoid coffee and smoking for an hour before training.

### 4.6. Sample Preparation

Study material (venous blood) was collected from patients twice (Monovette blood collection kit), at the beginning of the study and after 3 months. A total of 90 samples were obtained (2 × 45 samples). Collection was performed in the morning, before the subjects ate breakfast, between 7:00 and 8:00 a.m. Collected samples were transported (as directed) in a transporter to the laboratory.

Serum samples collected from patients were stored in −80 °C until analysis. Frozen plasma samples were thawed at 4 °C. Prior to extraction, samples were pooled within their respective experimental groups by combining equal aliquots from 3 individual samples. For this, 100 μL was extracted with 300 μL of a cold mixture of methanol and ethanol (1:1, *v*/*v*, −20 °C, both Optima LC-MS grade, Thermo Fisher Scientific, Waltham, MA, USA). Samples were then vortexed in Eppendorf tubes (30 s), cooled for 15 min (−20 °C), and centrifuged at 4 °C at 16,000 *g* (10 min). The obtained supernatant was filtered through a nylon filter (0.2 μm, 4 mm, Thermo Fisher Scientific). The extracts placed in inserts were then subjected to LC-MS analyses [20].

### 4.7. Mass Spectrometry Analysis

Formic acid (LC–MS grade) was obtained from Merck (Darmstadt, Germany). Acetonitrile and methanol (both Optima LC-MS grade) were purchased from Thermo Fisher Scientific (Waltham, MA, USA). Ultra-pure water was obtained from a water purification system (Millipore Direct-Q3-UV, Merck, Darmstadt, Germany).

The analysis was performed on a high-performance liquid chromatograph (HPLC, 1290 Infinity, Agilent Technologies, Santa Clara, CA, USA) coupled with a 6550 iFunnel Q-TOF mass spectrometer (Agilent Technologies) equipped with an ion source (Jet Stream Technology, Agilent Technologies) operated in positive mode (ESI+). The samples were separated using an RRHT Zorbax Extend C18 column (2.1 × 100 mm, 1.8 μm, Agilent Technologies). The mobile phase consisted of 0.1% formic acid in water (A) and 0.1% formic acid in acetonitrile (B) with a flow rate of 0.4 mL/min. Elution was performed for 30 min with a 2 min post run at 3%B. The gradient elution conditions were 0–25 min, 3%B to 95%B; 25–30 min, 95%B. The injection volume was 5 μL, and the column temperature was 45 °C. The ion source (nitrogen) temperature was 225 °C; flow rate: 12 L/min; nebulizer pressure: 50 psi; sheath gas temperature: 275 °C; sheath gas flow: 12 L/min; and capillary voltage: 3500 V. The nozzle voltage was set at 1000 V and the fragmentor voltage at 275 V; the mass scan mode range was *m*/*z* 100–1700. Internal mass calibration was enabled using two reference masses at *m*/*z* 121.0509 and *m*/*z* 922.0098. MS scans were acquired using Agilent MassHunter Data Acquisition software (B.09.00), and processing was performed using Agilent MassHunter Qualitative analysis software (B.10.00).

### 4.8. Data Processing and Statistical Analysis

Raw mass spectrometry data were processed with Molecular Feature Extractor in Agilent Mass Hunter Qualitative Analysis Software with limits of the used peak height set to 600 counts, compound absolute count set to 50,000 counts, and quality score ≥ 80. The resulting data containing mass-to-charge ratios (*m*/*z*), retention time (RT), and peak height were converted to CEF format prior to Mass Profiler Professional 15.1 (MPP Agilent Technologies, Santa Clara, CA, USA) software import. All features were first aligned using a 0.1% + 0.15 min RT window and 5 ppm + 2.0 mDa mass window. The metabolites were screened using the Metlin XCMS database with isotope alignment in the range 90–100%.

The statistical analysis and visualization were performed with RStudio software (ver. RStudio 2021.09.0 + 351 “Ghost Orchid” Release). The libraries used for analyses were as follows: dplyr, tidyr, ggplot2, car, rstatix, and multicompview. Missing data and insufficient levels for statistical analysis were handled by filtering compounds. Pairwise comparisons were performed for each compound using Dunn’s test with Holm’s adjustment for multiple comparisons. A heatmap was generated to display adjusted *p*-values (*p*.adj) for pairwise comparisons for compounds with significant differences. The heatmap color-coded *p*-values, highlighting significant results (*p* < 0.05) to identify meaningful differences across conditions.

Boxplots were created for each compound to display the distribution of concentration. Statistical significance from Dunn’s test was annotated on the plots using asterisks (*, **, ***) to indicate the level of significance (*p* < 0.05, 0.01, and 0.001, respectively).

## 5. Conclusions

Metabolomics demonstrates that schizophrenia involves systemic metabolic disturbances affecting energy, amino acid, lipid, and redox pathways. The robust findings strengthen the case for metabolic involvement, but specificity is limited, medication effects are confounding, and most findings remain correlational. Future progress requires multi-center, longitudinal metabolomics integrated with genomics, proteomics, imaging, and microbiome studies. Ultimately, metabolomics will likely support multimodal biomarker panels for precision psychiatry [51,52,53].

## Figures and Tables

**Table 1:** Initial (T1) parameters for NF, REH, and CON groups.

Variable	NF		REH		CON		ANOVA	
	M	SD	M	SD	M	SD	F	*p*
Age (years)	39.07	7.41	37.00	7.03	36.33	7.28	0.58	0.564
Education (years)	11.93	3.06	12.67	2.06	12.80	2.08	0.85	0.433
BMI (kg/m^2^)	28.09	3.18	28.59	3.17	30.68	3.43	0.13	0.880
PANSS Positive	17.27	3.61	15.13	1.55	15.67	2.77	2.40	0.103
PANSS Negative	24.67	5.21	23.00	3.38	23.20	4.23	0.66	0.522
PANSS General	37.20	5.72	41.47	8.89	38.20	7.99	1.28	0.290
PANSS Total	79.13	9.42	79.60	11.84	78.60	10.79	0.03	0.968
Diagnosis (years)	10.47	5.17	9.80	4.23	11.13	5.57	0.27	0.769
Antipsychotics (olanzapine equivalents in milligrams)	18.77	5.35	18.83	4.78	20.00	6.55	0.23	0.796

NF—neurofeedback group, REH—rehabilitation group, CON—control group, PANSS—Positive and Negative Syndrome Scale (Positive, Negative, General, Total), BMI—body mass index, M—mean, SD—standard deviation, ANOVA—F Statistics, 3-group comparison, *p*—*p*-value significance at *p* < 0.05.

**Table 2:** PANSS results of 3-month therapy (T1 vs. T2) in NF, REH, and CON groups of patients.

Group	Subtest	T1		T2		F	*p*
		M	SD	M	SD		
NF	Positive	17.27	3.61	13.33	1.59	14.89	0.001
	Negative	24.67	5.21	19.20	2.48	13.48	0.001
	General	37.20	5.72	31.33	11.72	3.04	0.093
	Total	79.13	9.42	63.87	13.44	12.99	0.001
REH	Positive	15.13	1.55	8.60	1.96	102.64	0.000
	Negative	23.00	3.38	11.60	2.56	108.47	0.000
	General	41.47	8.89	27.33	3.92	31.75	0.000
	Total	79.60	11.84	47.53	6.00	87.52	0.000
CON	Positive	15.67	2.77	15.13	2.53	0.30	0.586
	Negative	23.20	4.23	22.07	4.35	0.52	0.475
	General	38.20	7.99	37.73	6.16	0.683	0.416
	Total	78.60	10.79	74.93	8.58	1.061	0.312

NF—neurofeedback group, REH—rehabilitation group, CON—control group.

**Table 3:** Replicated metabolomic alterations in schizophrenia with main references.

Pathway/Class	Metabolite (s)	Direction (↑/↓)	Notes	Main References
Energy metabolism	Lactate	↑	Cognitive impairment, mitochondrial dysfunction	[3]
Energy metabolism	Pyruvate	↑	Altered glycolysis	[2,10]
Energy metabolism	Glucose-6-phosphate	↑	Biomarker panels	
Tryptophan–Kynurenine	Kynurenine	↑	Immune activation link	[12]
Tryptophan–Kynurenine	Kynurenic acid	↑	Elevated in FEP, NMDA receptor antagonist	[4]
Arginine/nitric oxide (NO) metabolism	Arginine	↓	Meta-analytically robust	[5]
Redox	Glutathione (GSH)	↓	Strong replication	[7]
Redox	Oxidized glutathione (GSSG)	↑	Predictive of treatment response	[14]
Lipidomics	Phospholipids/Sphingolipids	↓	Membrane and myelin disruption	[6,11]
Other	Cortisol	↑	Correlated with symptoms	[13]

↑—increase, ↓—decrease.

## Data Availability

No new data were created or analyzed in this study. Data sharing is not applicable to this article.

## Supplementary Materials

The following supporting information can be downloaded at: https://www.mdpi.com/article/10.3390/ijms27010380/s1.
