# Supplementary material for: Metabolic Plasticity in Schizophrenia: Clinical Rehabilitation Meets LC–MS Metabolomics and Neurofeedback
Source: Int J Mol Sci. 2025 Dec 29;27(1):380. doi: 10.3390/ijms27010380 (PMC12785504; doi:10.3390/ijms27010380)
Supplement: Supplementary file 1 [file ijms-27-00380-s001.zip › ijms-3942297-supplementary.pdf]

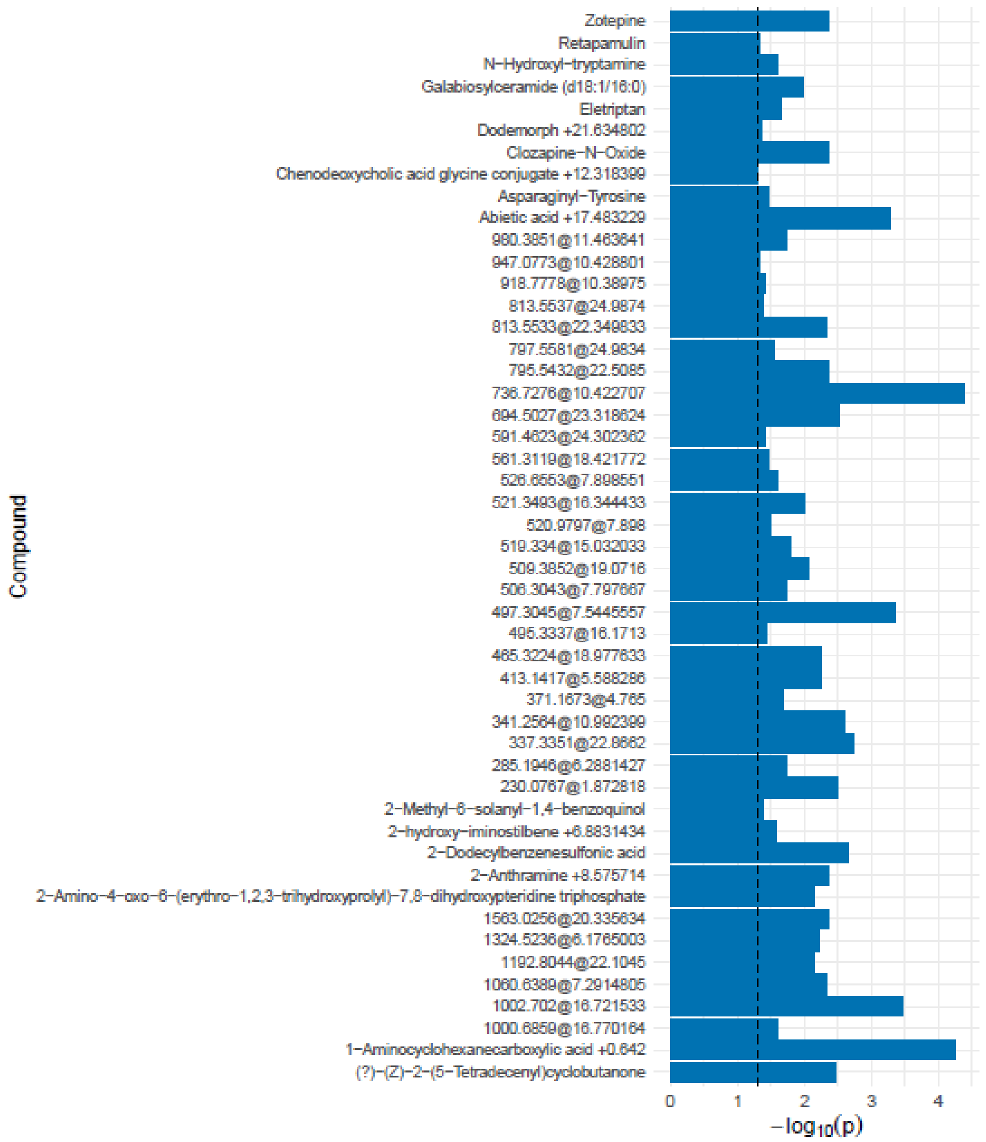

Figure S1. All significant features differentiating, based on the time effect of groups REH, NF and CON from two-way ANOVA. Horizontal bars represent $-\log_{10}$(p-values) for each compound. The vertical dashed line indicates the threshold for statistical significance ($p = 0.05$).

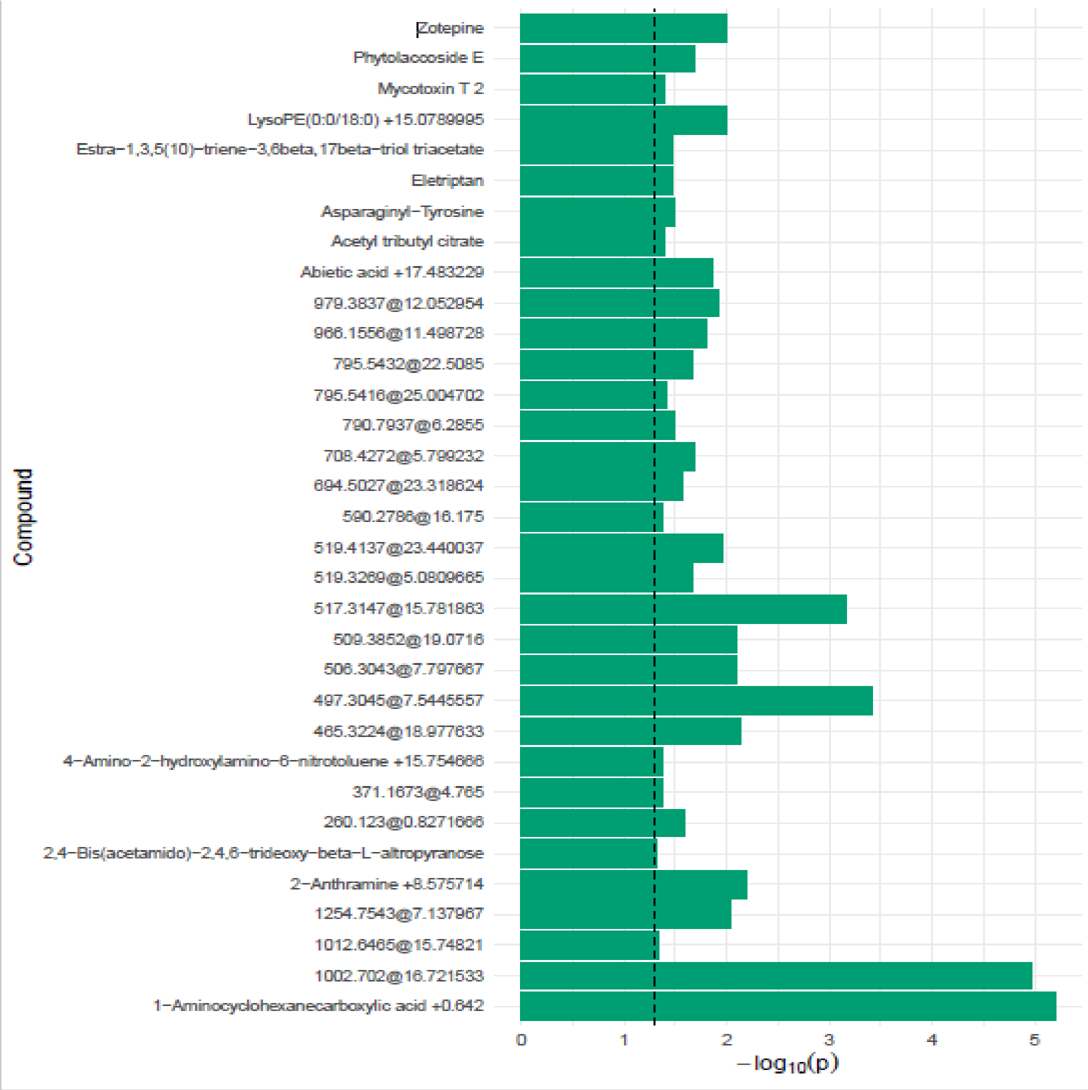

Figure S2. All significant features differentiating, based on combined time and group interactions of groups REH, NF and CON from two-way ANOVA. Horizontal bars represent $-\log_{10}$(p-values) for each compound. The vertical dashed line indicates the threshold for statistical significance ($p = 0.05$).

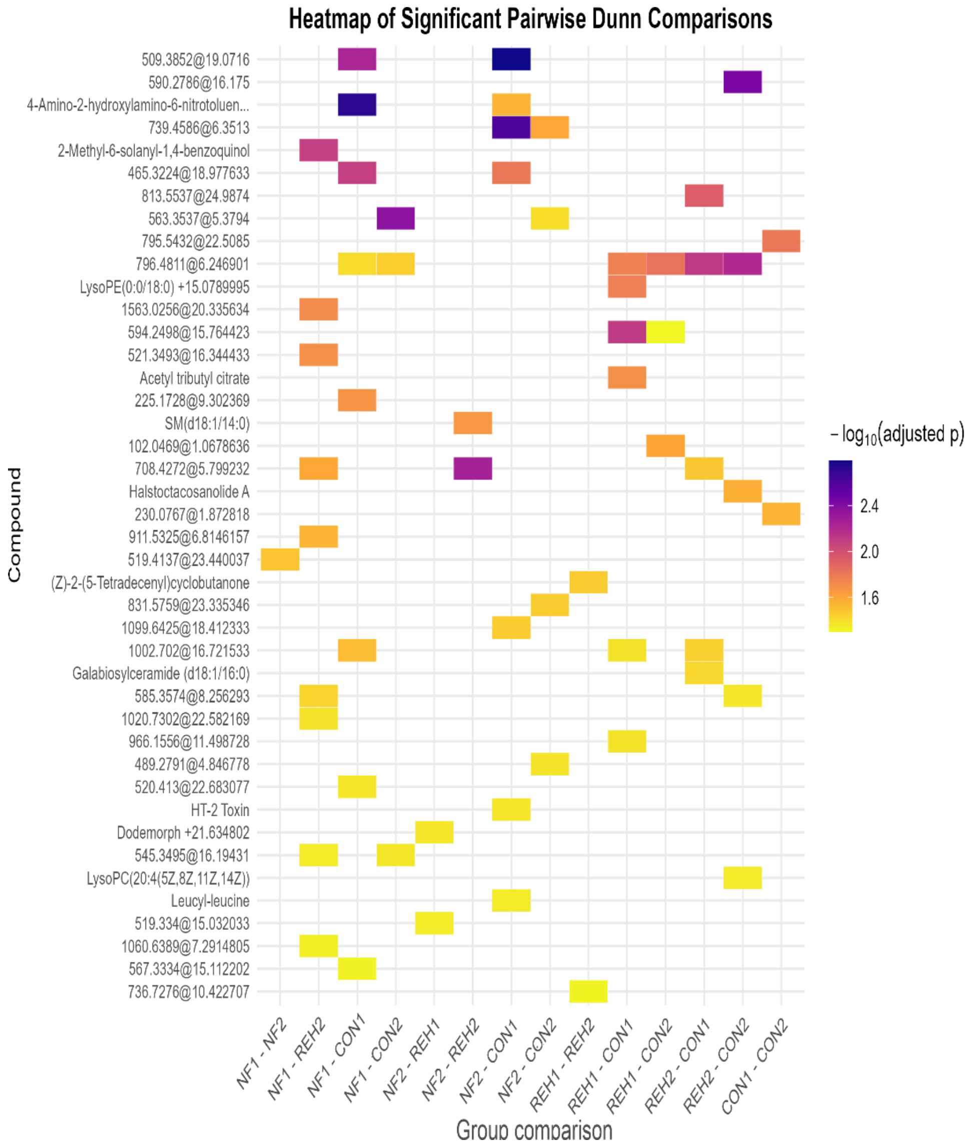

Figure S3. Heatmap displaying statistically significant pairwise group comparisons for all individual features, as identified using Dunn's post-hoc test with Holm correction for multiple testing (*adjusted* $p < 0.05$).
